# Genetic Variation of HPV53 and the Identification of T-Cell Epitopes

**DOI:** 10.3390/microorganisms14071395

**Published:** 2026-06-24

**Authors:** Li Wang, Sudan Jiao, Sihan Lan, Yuxiao Zhang, Jing Yu, Jie He, Hongping Zhang, Min Feng

**Affiliations:** 1 Institute of Medical Biology, Chinese Academy of Medical Sciences & Peking Union Medical College, Kunming 650118, China; s2023018018@pumc.edu.cn (L.W.); jiaosd2003@163.com (S.J.); celeste_3@tju.edu.cn (S.L.); 25110700149@m.fudan.edu.cn (Y.Z.); 2 Peking University Cancer Hospital Yunnan, Yunnan Cancer Hospital, The Third Affiliated Hospital of Kunming Medical University, Kunming 650118, Chinahejieagz@163.com (J.H.)

**Keywords:** HPV53, clinical infection profiles, phylogenetic analysis, genetic variation, T-cell epitopes

## Abstract

Human papillomavirus type 53 (HPV53) is one of the most prevalent HPV genotypes in China, frequently detected in cervical intraepithelial neoplasia and cervical cancer, yet remains outside the coverage of all currently available prophylactic vaccines and is relatively understudied. This study performed a comprehensive analysis of HPV53 clinical infection profiles, genomic diversity, and T-cell epitopes to inform therapeutic vaccine development. Clinical analysis of 158 HPV53-positive patients showed that infections were most prevalent in women aged 40–59 years, with persistent infection identified in 13.3% participants and a subset of cases associated with cervical lesions. Genomic analysis of 134 HPV53 isolates identified four lineages (A-D, with lineage D further subdivided into four sublineages, and an overall nucleotide variability of 4.4%. E2 was the most variable protein while E7 was the most conserved. Immunoinformatic prediction identified 176 HLA class I-restricted T-cell epitopes across E6, E7, E1, and E2, from which 20 candidates were selected for experimental validation. Ten demonstrated strong HLA binding affinity in vitro, and murine immunization identified a E6 peptide VYNFAYTDL as an immunodominant epitope. Three validated epitopes exhibited sequence overlap with 12 to 13 of other 13 high-risk HPV genotypes, suggesting their potential as broadly cross-reactive targets. These findings clarify the genomic diversity and immunogenic epitope landscape of HPV53, providing a foundation for the rational design of therapeutic vaccines.

## 1. Introduction

Human papillomaviruses (HPVs) are a diverse group of epitheliotropic, double-stranded DNA viruses. Persistent infection with high-risk HPV genotypes is the principal etiological factor for cervical cancer, as well as other anogenital and oropharyngeal malignancies [1]. To date, six prophylactic HPV vaccines have been successfully implemented worldwide, significantly reducing infections caused by a maximum of nine HPV genotypes (HPV6, 11, 16, 18, 31, 33, 45, 52, and 58) [2]. However, these vaccines neither eliminate established infections nor treat existing HPV-associated lesions [3]. Moreover, their protective scope remains limited, as they fail to cover all high-risk and probable high-risk HPV genotypes. Therefore, the development of broadly protective prophylactic vaccines, as well as therapeutic vaccines capable of eliciting effective cellular immune responses against HPV-infected and transformed cells, remains a critical and unmet medical need.

Epidemiological studies have revealed marked geographic heterogeneity in HPV genotype distribution [4]. Several high-risk and probable high-risk HPV genotypes not covered by existing vaccines remain highly prevalent [5], raising concerns about their potential contribution to cervical carcinogenesis. Among these, HPV53 has emerged as one of the most frequently detected HPV genotypes in China. Multiple large-scale epidemiological studies have consistently reported a high prevalence of HPV53 in Chinese women [6,7,8,9], and this genotype has also been frequently detected in cases of cervical intraepithelial neoplasia and cervical cancer [10,11]. This sustained high infection rate suggests that HPV53 may contribute more substantially to the cervical disease burden in China than is currently recognized. Despite its epidemiological relevance, HPV53 remains comparatively understudied relative to other high-risk genotypes, and comprehensive investigations into its genetic diversity and immunological characteristics are still limited.

Genetic variation is a fundamental feature of HPV evolution with important implications for viral persistence, pathogenicity, and immune evasion [12]. Variants arising in early open reading frames (ORFs) can modulate host immune responses by influencing antigen presentation, major histocompatibility complex (MHC)-peptide binding affinity, and T-cell recognition [13,14,15]. Such variation has direct implications for immune recognition, as cytotoxic T lymphocyte (CTL)-mediated responses—central to both viral clearance and the rationale for therapeutic vaccination—depend critically on epitope conservation across circulating variants. Systematic characterization of HPV53 genetic variation is essential for identifying conserved, immunologically relevant regions suitable as targets for therapeutic vaccine design.

Cell-mediated immunity, particularly CD8^+^ CTL responses, plays a pivotal role in controlling HPV infection and driving regression of HPV-associated lesions [16,17]. Therapeutic HPV vaccines are designed to elicit robust virus-specific T-cell responses by delivering viral antigens via platforms such as viral vectors or nucleic acid-based systems to antigen-presenting cells, enabling the specific recognition and elimination of HPV-infected and transformed cells [18,19]. Recent advances in immunoinformatics have enabled the computational prediction of T-cell epitopes based on MHC binding affinity, antigen processing efficiency, and population coverage, though experimental validation remains indispensable for confirming the immunogenicity of candidate epitopes.

In this study, HPV53 was selected as the primary focus due to its high prevalence among Chinese women and the relative paucity of existing research on this genotype. Using HPV53-positive clinical samples, we first performed an analysis of the clinical characteristics associated with HPV53 infection. Complete HPV53 genomes were subsequently obtained from these samples and, together with publicly available sequence data, used to conduct a comprehensive characterization of HPV53 genetic variation, with particular emphasis on mutation profiles within the early open reading frames E6, E7, E1, and E2. Building upon these findings, immunoinformatic tools were employed to predict potential T-cell epitopes restricted by globally and regionally prevalent HLA class I alleles. The binding affinities of selected candidate epitopes to MHC molecules were subsequently validated experimentally, and their capacity to elicit HPV53-specific T-cell responses was further evaluated in a mouse model.

## 2. Materials and Methods

### 2.1. Clinical Specimens and Whole-Genome Sequencing

Between May 2022 and January 2026, a total of 158 cervicovaginal samples were collected from patients who attended the gynecological clinic of the Affiliated Hospital of Kunming Medical University for HPV screening and were confirmed as HPV53-positive. The key screening procedures involved cervical exfoliated cell collection, DNA extraction, and universal fluorescence PCR-based HPV genotyping, which was performed using a Human Papillomavirus Genotyping Kit for 23 Types (Hybribio Biotech Co., Ltd., Guangdong, China). This qualitative assay concurrently identifies 23 HPV genotypes, encompassing 17 high-risk types (HPV 16, 18, 31, 33, 35, 39, 45, 51, 52, 53, 56, 58, 59, 66, 68, 73, and 82) and 6 low-risk types (HPV 6, 11, 42, 43, 44, and 81). This study was reviewed and approved by the Ethics Committee of the Affiliated Hospital of Kunming Medical University (KYCS2022024). Written informed consent was obtained from all patients prior to sample collection. Relevant clinical data, including age, colposcopy referral status, and histopathological findings, were obtained from the medical records. All data were anonymized and did not include any personally identifiable information.

HPV53 DNA was extracted using the QIAamp DNA Mini Kit (Qiagen, Hilden, Germany, Cat# 51304) and amplified by rolling circle amplification (Cytiva, Marlborough, MA, USA, Cat# 25-6400-10). Full-length viral genomes were amplified in three overlapping fragments using type-specific primers (Table 1) and PrimeSTAR Max DNA Polymerase (Takara, Kusatsu, Japan, Cat# R045A). PCR products were verified by gel electrophoresis and subsequently sequenced by Sanger sequencing using a primer-walking strategy. The resulting sequences were aligned and assembled using SnapGene software (version 6.0.2) to obtain complete full-length genome sequences.

### 2.2. NCBI GenBank Sequence Acquisition

To augment the dataset and maximize the representation of HPV53 genetic diversity, 55 full-length HPV53 genomes available up to January 2026 were retrieved from GenBank (https://www.ncbi.nlm.nih.gov/genbank/, accessed on 31 January 2026) (Table S1). This dataset comprised the HPV53 reference strain (accession number: X74482) and 54 additional variant genomes, facilitating a more comprehensive genetic analysis alongside the novel isolates obtained in this study.

### 2.3. Novel Variants Identification and Recombination Detection

Full-length HPV53 sequences obtained from the clinical samples and GenBank were aligned via MUSCLE (version 3.8.1551) to identify novel variants. After excluding duplicates, potential recombination events were screened in RDP4 (version 4.101) using seven algorithms (RDP, GENECONV, BootScan, MaxChi, Chimaera, SiScan, 3Seq). Subsequently, any detected recombination signals were verified using SimPlot (version 3.5.1).

### 2.4. Evolutionary Analyses and Phylogenetic Tree Construction

Sequences showing evidence of recombination were excluded from phylogenetic tree construction. Phylogenetic reconstruction was conducted in MEGA 11 using Maximum Likelihood method based on the Kimura 2-parameter (K2P) model with 1000 bootstrap replications. The K2P model was selected because it was widely used in papillomavirus phylogenetic studies and was considered appropriate for estimating evolutionary distances among closely related HPV variants. The resulting phylogenetic tree was visualized with the iTOL online tool (https://itol.embl.de/, accessed on 2 June 2026).

A total of 89 complete HPV53 genome sequences from clinical samples were aligned against the prototype sequence (GenBank association number: X74482) to identify nucleotide and amino acid variations using MEGA 11. The revised prototype genome sequence of HPV53, previously reported to correct probable errors in the original prototype clone, was used as the reference sequence throughout [20]. Selection pressure analyzes were done using Fixed Effects Likelihood (FEL) tool implemented online on Datamonkey server (https://www.datamonkey.org/, accessed on 2 June 2026) with a cutoff *p*-value of 0.05.

### 2.5. T-Cell Epitopes Prediction

T-cell epitopes within the HPV53 early proteins (E6, E7, E1 and E2) of the reference strain were predicted using the Immune Epitope Database (IEDB) (http://tools.iedb.org/main/tcell/, accessed on 3 September 2025) with recommended methods [21]. HLA class I (HLA-I) alleles with relatively high population frequency in both Chinese [22] and global populations [23] were selected for analysis (Table S2), such as HLA-A*02:01, A*11:01, and A*24:02. Candidate peptides were identified based on the following criteria: peptide length of 8–14 amino acids, percentile rank (PR) < 2.0, VaxiJen (http://www.ddg-pharmfac.net/vaxijen/VaxiJen/VaxiJen.html, accessed on 10 September 2025), antigenicity score > 0.4, IEDB total score > −1.5, and MHC binding affinity (IC_50_) < 500 nM. Peptides demonstrating high sequence conservation across all analyzed isolates were prioritized for further experimental validation. Subsequently, to account for genomic diversity, the impact of non-synonymous mutations identified across different lineages and sublineages on the binding affinities of these candidate epitopes was systematically evaluated.

### 2.6. Synthetic Peptides

All peptides were chemically synthesized by Sangon Biotech (Shanghai, China) with ≥95% purity. Peptides were dissolved in DMSO, diluted in PBS, and stored at −20 °C until use.

### 2.7. Peptide Exchange ELISA Assay

Recombinant HLA-I monomers (HLA-A*02:01, -A*11:01, -A*24:02, and -B*07:02) complexed with the UV-cleavable peptides were purchased commercially (BioLegend, San Diego, CA, USA, Cat# 280003, 280007, 280019, and 280009). For the exchange reaction, 20 μL of each candidate peptide (diluted to 50 μM in PBS) and 20 μL of monomer (25 μg/mL) were combined in a 96-well V-bottom plate. To initiate peptide dissociation, the mixture was exposed to UV light (366 nm) for 30 min on ice, followed by incubation at 37 °C for 30 min in the dark to facilitate target peptide binding. Exchange efficiency was subsequently quantified using the LEGEND MAX™ Flex-T™ Human Class I Peptide Exchange ELISA Kit (BioLegend, San Diego, CA, USA, Cat# 447207) according to the manufacturer’s instructions. Specific control peptides (Table S3) were used as positive and negative controls to validate the assay.

### 2.8. Peptide/HLA-A2 Binding Assay

Binding affinity of candidate peptides to HLA-A*02:01 was assessed using a TAP-deficient T2 cell-based stabilization assay. The T2 cell line was kindly provided by Prof. Li Shi (IMBCAMS). Cells were seeded in 24-well plates (1 × 10^6^ cells/well) and incubated with individual candidate peptides (200 μg/mL) in the presence of β2-microglobulin (3 μg/mL; ProSpec, Rehovot, Israel, Cat# pro-553) at 37 °C with 5% CO_2_ for 18 h. Cells were then washed twice with cold PBS containing 1% BSA and stained with a FITC-conjugated anti-HLA-A2 antibody (BioLegend, Cat# 343303) for 30 min at 4 °C in the dark. Surface HLA-A2 expression was analyzed by flow cytometry and the relative peptide binding affinity was expressed as the fluorescence index (FI), calculated as: FI  =  (mean FITC fluorescence with the given peptide—mean FITC fluorescence without peptide)/mean FITC fluorescence without peptide.

### 2.9. Mice Immunization and Splenocyte Isolation

Female C57BL/6 mice (6–8 weeks old), purchased from Beijing Vital River Laboratory Animal Technology Co., Ltd., Beijing, China, were housed under specific pathogen-free (SPF) conditions in the barrier system at the Central Animal Service Center of Institute of Medical Biology, Chinese Academy of Medical Sciences (IMBCAMS). Experimental protocols were approved by the IMBCAMS Laboratory Animal Ethics Committee (DWSP202311008).

Peptides were pooled according to their source proteins, yielding four distinct peptide pools corresponding to E6, E7, E1, and E2. Mice (*n* = 5 per group) were immunized intramuscularly under anesthesia three times at one-week intervals. Each immunization consisted of 200 μg of the protein-specific peptide pool formulated with 20 μg of CpG ODN1826 adjuvant (synthesized by Sangon Biotech, Shanghai, China). Control groups received either CpG ODN1826 alone or PBS. One week after the final immunization, mice were sacrificed, and spleens were harvested and homogenized. The resulting cell suspension was then filtered through a 40 µm cell strainer. Erythrocytes were depleted using Red Blood Cell lysing buffer (Miltenyi Biotec, Bergisch Gladbach, Germany, Cat# 130-094-183). Splenocytes were then washed and resuspended in RPMI 1640 medium supplemented with 10% fetal bovine serum (FBS) for subsequent assays.

### 2.10. Detection of Peptide-Specific T Cells by IFN-γ ELISpot

Splenic lymphocytes (1 × 10^6^) were seeded in 24-well plates and stimulated with 20 μg of HPV53 epitope pools or individual peptides at 37 °C with 5% CO_2_ for 24 h. PBS and PMA served as negative and positive controls, respectively. Following initial stimulation, pre-activated cells (1 × 10^5^/100 μL/well) were transferred to pre-coated ELISpot 96-well plates (Mabtech, Stockholm, Sweden, Cat# 3321-4HST-2) and incubated for an additional 24 h. Interferon-γ (IFN-γ) production was assessed according to the manufacturer’s instructions. The number of spot-forming cells (SFCs) was quantified using an automated ELISpot image analyzer (Cellular Technology Limited, CTL, Cleveland, OH, USA).

### 2.11. Homology Analysis of Identified Epitopes

To assess sequence conservation, the amino acid sequences of validated dominant T-cell epitopes were aligned with corresponding sequences from 13 high-risk HPVs (HPV16, 18, 31, 33, 35, 39, 45, 51, 52, 56, 58, 59, and 68) using the NCBI BLASTp tool (https://blast.ncbi.nlm.nih.gov/Blast.cgi, accessed on 15 March 2026). The percentage of amino acid identity between HPV53-derived epitopes and their counterparts in other high-risk genotypes was subsequently calculated.

### 2.12. Statistical Analysis

All statistical analyses were performed using GraphPad Prism 7. Data are presented as the mean ± standard deviation (SD). Homogeneity of variances was evaluated prior to analysis. Where variance heterogeneity was detected, group comparisons were conducted using Brown–Forsythe one-way ANOVA followed by Dunnett’s T3 post hoc test. Comparisons between two independent groups were performed using unpaired Student’s *t*-tests. A *p* value < 0.05 was considered statistically significant.

## 3. Results

### 3.1. Clinical Characteristics and HPV53 Infection Profiles

**Figure 1:**
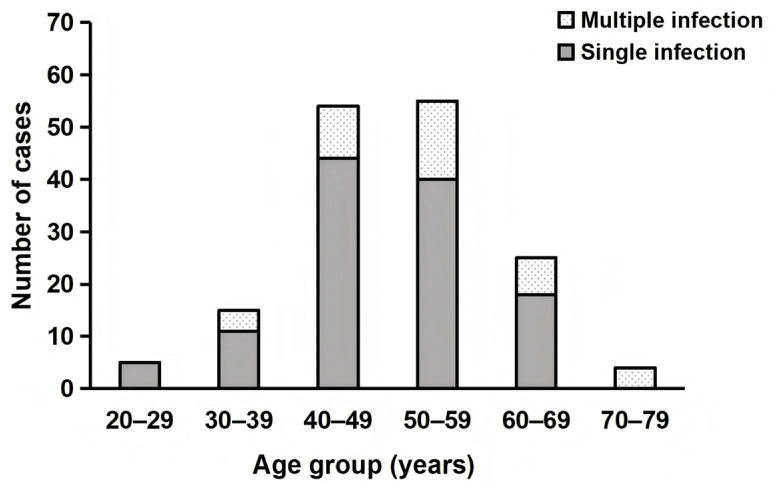
Age-specific prevalence of HPV53 infection.

A total of 158 HPV53-positive patients (aged 22–78 years) were included in this analysis (Table S4). When patients were stratified into 10-year age intervals, the highest prevalence of HPV53 was observed in the 50–59-year and 40–49-year age groups, accounting for 34.8% and 34.2% of the cohort, respectively (Figure 1). Notably, the 50–59-year age group exhibited the highest frequency of multiple HPV infections.

Regarding infection patterns, HPV53 was detected as a single-type infection in the majority of cases (74.7%, 118/158), while co-infections were observed in the remaining 40 cases (25.3%). The most frequently detected co-infecting HPV types were HPV52, HPV58, HPV39, and HPV51.

Persistent HPV53 infection was identified in 21 participants, defined as testing positive for HPV53 in two or three consecutive screenings at 3–24-month intervals. Within this subgroup, five patients presented with co-infections (specifically with HPV58, HPV68, or HPV81), while the remaining 16 cases exhibited persistent single-type infections.

Among the 158 patients, 52 were referred for colposcopy and 50 underwent directed biopsy. Histopathological analysis showed that precancerous lesions (LSIL or HSIL) accounted for 13.3% (21/158) of the cohort. Among these patients, single HPV53 infection was observed in 13 cases; multiple HPV infections were identified in the remaining 8 cases, and detailed information on the co-infection HPV genotypes can be found in Table S4. Notably, one patient with single HPV53 infection was diagnosed with invasive cervical cancer.

### 3.2. Genomic Diversity of HPV53 Variants

A total of 102 complete HPV53 genomes were successfully characterized from clinical specimens. After removing duplicates, 89 novel full-length sequences were identified and deposited in GenBank (accession numbers PX685428-PX685504 and PZ161758-PZ161769; Table S4). Genome-wide analysis revealed an overall nucleotide variability of 4.4% (342/7859), with a maximum pairwise nucleotide diversity of 1.7% across complete genomes, observed between isolates 53YN23 and 53YN54. Within the six ORFs, 111 of 2476 amino acid sites (4.5%) were variable (Table 2). The non-coding upstream regulatory region (URR) exhibited higher variability than the coding regions, while E2 was the most diversified protein with overall amino acid differences of 9.1%.

**Figure 2:**
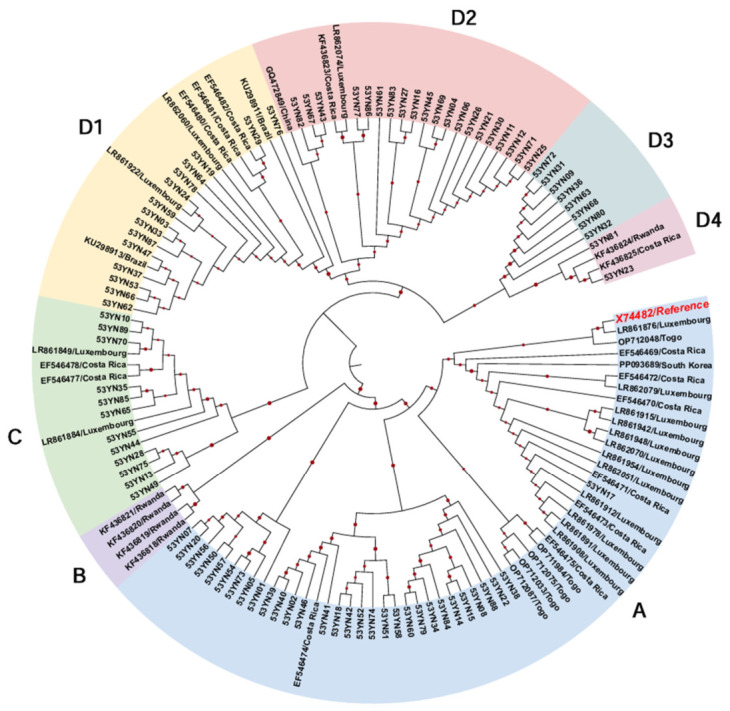
Phylogenetic tree of HPV53. A maximum likelihood (ML) phylogenetic tree was constructed using MEGA 11. The reference sequence (strain X74482/Reference) is highlighted in bold red. The red circles indicate the bootstrap values that are >75%.

Recombination analysis identified a putative recombination event in strain 53YN48. RDP4 detected a potential recombinant fragment spanning nucleotide positions 1234–3586, with 53YN15 and EF546481 inferred as the major and minor parental sequences, respectively. The recombination signal was further validated by SimPlot analysis, which revealed a shift in sequence similarity within the corresponding genomic region (positions 1221–3581) (Figures S1 and S2). To avoid potential bias in phylogenetic inference, strain 53YN48 was excluded prior to phylogenetic tree construction. To assess evolutionary relationships, the 88 novel isolates were analyzed together with 46 variants and the HPV53 prototype obtained from GenBank (Tables S1 and S4). Phylogenetic topology based on complete genome alignment clustered HPV53 variants into four distinct lineages, designated A to D (Figure 2). Mean inter-lineage nucleotide divergence ranged from 1.54 to 1.81%, with maximum pairwise nucleotide divergence (1.9%) observed between lineages B and D. Lineages C and D were more closely related, with mean pairwise differences of 0.76–0.91%. Lineage D was further subdivided into four sublineages (D1–D4), with mean pairwise differences ranging from 0.26 to 0.46% (Table S5). The 88 isolates from the present study were distributed across all lineages except lineage B. Sublineage D3 consisted exclusively of eight sequences identified in this study. Isolate 53YN32 (a D3 variant) exhibited an intermediate mutational profile that may bridge the evolutionary transition between sublineages D2 and D3, carrying several D2-specific nucleotide changes (e.g., E6-G129C; E1-A1605G; L1-G6356A and -G6441T) while lacking mutations conserved in D3 (e.g., E1-A1233C, -T1947C, -C1984T and E2-T2959C, -C3869T). Lineage and sublineage-specific nucleotide variations were determined across the 88 genomes, with 24 single nucleotide polymorphisms (SNPs) for lineage A, 28 for lineage B, and 19 for lineage D. For example, A1363C and T3217C were unique to lineage C, whereas A112G and C256A were specific to lineage D. In addition, lineage A harbored a TGGG deletion at position 7533 in the URR (Table S6).

A total of 342 nucleotide changes were identified across the 89 isolates, with the number of SNPs per genome ranging from 10 (53YN17) to 128 (53YN24) (Table S6). Non-synonymous mutations accounted for 31.3% (107/342) of all changes and were detected in all 89 sequences. Several amino acid variants were conserved across all lineages, including T77I in E1, as well as N25A, S149P, S153N, and V340A in E2. Additionally, specific nucleotide positions exhibited multiple mutation patterns resulting in either identical or distinct amino acid substitutions. For instance, at nucleotide position 3814 in E2, substitution of a cytosine (C) with either thymine (T) or guanine (G) produced synonymous changes. In contrast, at position 6945 in L1, a C-to-T substitution resulted in a proline (P) to serine (S) change, while a C-to-A substitution led to a proline (P) to threonine (T) change.

### 3.3. T-Cell Epitope Prediction and Experimental Validation: Binding Capacity and Immunogenicity

A total of 176 potential HLA class I–restricted T-cell epitopes were identified across the HPV53 early proteins, comprising 39 in E6, 17 in E7, 54 in E1, and 66 in E2 (Table S7). The majority of predicted epitopes were located in E2, while the fewest were identified in E7. This distribution likely reflects the greater length and sequence diversity of E2 relative to E7, rather than differential immunogenic potential. For subsequent validation, epitopes restricted by four HLA-I alleles (HLA-A*02:01, -A*11:01, -A*24:02, and -B*07:02) were targeted based on their high population coverage in both China and globally. Applying a stringent PR threshold of < 1.0, 20 candidate epitopes with favorable predicted antigenicity and binding properties were shortlisted (Table 3).

**Figure 3:**
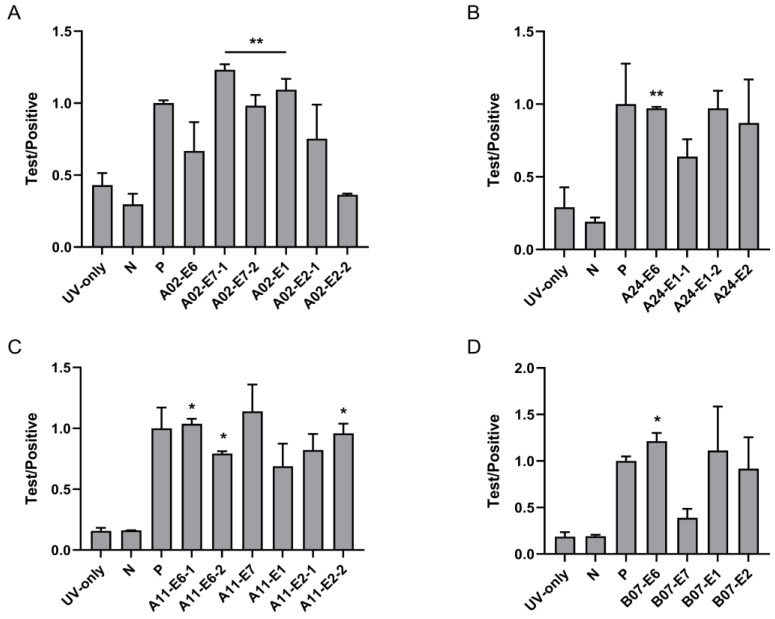
Validation of peptide-MHC binding affinity across diverse HLA-I alleles. Bar graphs showing the Test/Positive OD ratios for HLA-A*02:01 (**A**), HLA-A*24:02 (**B**), HLA-A*11:01 (**C**), and HLA-B*07:02 (**D**). OD values were normalized to the positive control (set as 1). Tested conditions include UV-only treatment, negative control (N), positive control (P), and candidate epitopes labeled according to their corresponding HLA allele and source protein. Data are presented as mean ± SD. * *p* < 0.05 and ** *p* < 0.01 vs. the corresponding negative control.

The 20 selected peptides were evaluated for binding capacity to four commercially available HLA-I monomers (HLA-A*02:01, -A*11:01, -A*24:02, and -B*07:02) using an ELISA-based peptide-exchange assay. Exchange efficiencies ranged from approximately 40% (A02-E2-2) to nearly 120% (A02-E7-1) relative to their respective positive controls (Figure 3). Several peptides exhibited strong binding to specific HLA alleles. For HLA-A*02:01, peptides A02-E7-1, A02-E7-2, and A02-E1 showed binding capacity comparable to or exceeding the positive control (Figure 3A). For HLA-A*11:01, peptide A11-E6-1, A11-E6-2, and A11-E2-2 showed robust binding (Figure 3C). For HLA-A*24:02 and HLA-B*07:02, peptide A24-E6 and B07-E6 showed significantly higher binding capacity, respectively; although A24-E1-2, A24-E2, B07-E1 and B07-E2 appeared to have elevated mean binding levels, these did not reach statistical significance compared to the negative control.

**Figure 4:**
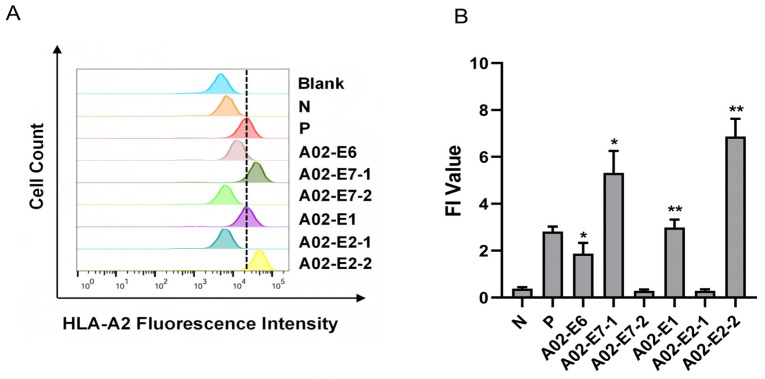
Assessment of peptide binding affinity to HLA-A*02:01 using a T2 cell-based assay. (**A**) Representative flow cytometry histograms showing HLA-A2 surface expression on T2 cells following incubation with different HLA-A2-restricted candidate peptides. Blank, unstained cells; N, negative control peptide; P, positive control peptide. (**B**) Quantification of peptide-induced HLA-A2 stabilization expressed as fluorescence index (FI), calculated from mean fluorescence intensity (MFI) values. * *p* < 0.05 and ** *p* < 0.01 vs. the corresponding negative control.

Binding of the six putative HLA-A*02:01-restricted epitopes was further assessed using a T2 cell-based stabilization assay. Four of the six peptides (67%) confirmed binding to HLA-A*0201 with varying affinities (Figure 4). Quantitative evaluation was conducted by calculating the FI based on the mean fluorescence intensity (MFI), with the positive peptide used as a reference (Figure 4B). Peptides A02-E7-1 and A02-E2-2 exhibited significantly higher FI than the positive control, and A02-E1 showed comparable FI values, collectively indicating strong binding capacity. Peptide A02-E6 demonstrated moderate binding activity. In contrast, A02-E7-2 and A02-E2-1 showed minimal increases in FI, with values approaching the negative control, indicating weak or negligible binding to HLA-A2.

**Figure 5:**
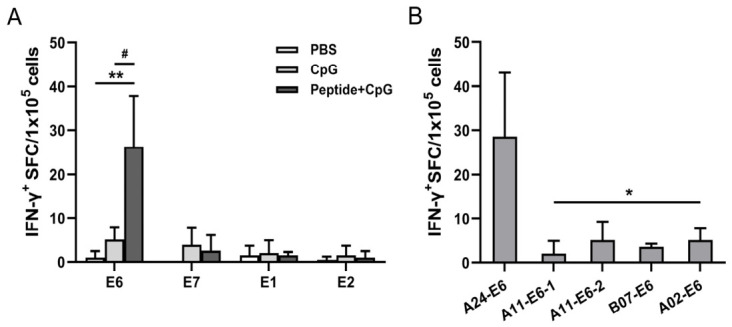
Identification of the immunodominant T-cell epitopes of HPV53 by IFN-γ ELISpot. (**A**) Splenocytes from mice immunized with PBS, CpG, or peptide plus CpG were stimulated in vitro with HPV53 E6, E7, E1, or E2 peptide pools. Data are shown as mean ± SD. ** *p* < 0.001 vs. the PBS control; # *p* < 0.001 vs. the CpG control. (**B**) Splenocytes from peptide-immunized mice were stimulated with individual E6-derived peptides. Data are shown as mean ± SD. * *p* < 0.05 vs. the A24-E6 peptide group.

For murine immunization, the 20 candidates were further refined according to murine MHC-related parameters, resulting in selection of 13 peptides from E6, E7, E1 and E2 for in vivo experiments (Table S8). IFN-γ ELISpot assays revealed that only the E6 peptide pool combined with CpG adjuvant induced a markedly higher number of IFN-γ-producing cells compared with the PBS- and CpG-immunized control groups (Figure 5A). Peptide pools derived from E7, E1, and E2 induced only low-level responses. To further identify the immunodominant epitopes within E6, individual E6-derived peptides were assessed. Distinct differences in IFN-γ spot numbers were observed among the tested peptides (Figure 5B). Notably, peptide A24-E6 induced a significantly stronger IFN-γ response than all other E6-derived peptides, while the remaining peptides elicited relatively weak or moderate responses. Collectively, these results indicate that T-cell responses elicited by HPV53 E6 are epitope-specific, with A24-E6 representing a dominant T-cell epitope.

### 3.4. Homology Analysis of Antigenic Epitopes Across 13 High-Risk HPV Types

To assess the potential breadth of cross-reactive immunity, homology analysis was conducted between the 10 experimentally validated epitopes and corresponding sequences from 13 high-risk HPV genotypes. Most validated epitopes shared sequence overlap with 5–8 of the 13 genotypes (Table S9). Notably, A02-E1 and A02-E2-2 showed homology with all 13 high-risk HPV types, and A24-E6 12, suggesting their potential as broadly cross-reactive targets. Among all epitopes, A11-E6-2 exhibited the highest degree of homology with HPV56, reaching 100% amino acid identity. At the HPV genotype level, HPV33, HPV51 and HPV52 exhibited relatively high homology with 9 of the 10 validated epitopes, whereas HPV45 shared homology with only 3, representing the most limited overlap observed.

## 4. Discussion

Persistent infection with oncogenic human papillomaviruses remains the principal cause of cervical cancer worldwide. Although currently available prophylactic vaccines have substantially reduced infections caused by several major HPV types, a number of high-risk or probable high-risk genotypes remain outside vaccine coverage. HPV53 has emerged as one of the most prevalent HPV genotypes in China, yet it remains entirely unaddressed by current prophylactic vaccines and has received comparatively little research attention. In the present study, we performed a comprehensive investigation of HPV53, encompassing clinical infection profiles, genome-wide genetic variation analysis, and the prediction and experimental validation of T-cell epitopes, with the aim of informing the rational design of HPV53-directed therapeutic vaccines.

The clinical data from 158 HPV53-positive patients revealed that infection was predominantly detected in women aged 40–59 years. This age distribution is consistent with the well-documented bimodal pattern of HPV prevalence, in which a second peak occurs among perimenopausal women [24,25]. Previous studies have shown that HPV infections in younger women are often cleared spontaneously through effective host immune responses [26], whereas middle-aged and older women exhibit higher rates of persistent infection and impaired viral clearance, thereby increasing the risk of cervical lesion development [27]. Notably, the 50–59 age group also exhibited the highest frequency of multiple infections, suggesting that immune senescence may contribute to susceptibility to co-infection in this demographic. These findings are broadly consistent with previous epidemiological reports from China documenting high HPV53 prevalence in perimenopausal women [7,8], and reinforce the need for continued surveillance of this genotype in older populations who fall outside the recommended age range for HPV vaccination.

Persistent HPV53 infection was identified in 13.3% of participants (21/158), and precancerous lesions were also detected in this cohort. Notably, one patient with single-type HPV53 infection was diagnosed with invasive cervical cancer. Despite the limited sample size, these observations align with previous reports implicating HPV53 in cervical intraepithelial neoplasia and malignancy [10,11], and support the oncogenic potential of HPV53. Sequence analysis revealed that HPV53 E6 and E7 proteins retain several key functional domains commonly found in highly oncogenic HPV types. These include the canonical zinc-binding domains within E6 [28]. In E7, the pRb-binding motif follows the L-X-C-X-E consensus pattern with natural amino acid variations, alongside a conserved C-terminal cysteine-rich zinc-binding domain [29], indicating that HPV53 shares the molecular framework required for oncogenic activity. On the other hand, compared with HPV16 and HPV18, substantial amino acid sequence divergence exists within the non-conserved regions of HPV53 E6 and E7. Such variations may alter their interactive dynamics with host tumor suppressor proteins (e.g., p53 and pRb), potentially modulating downstream biological activities distinct from those of the classical high-risk HPV types. Given the limitation in current direct functional and clinical evidence regarding HPV53, further investigations are warranted to fully elucidate its exact oncogenic capacity and pathogenic mechanisms. The detection of co-infections with HPV52, HPV58, HPV39, and HPV51 is also noteworthy, as co-infection with multiple high-risk genotypes has been associated with increased risk of cervical disease progression [30]. It should be mentioned that the clinical cohort was derived from a single center in Yunnan Province, China, and larger multicenter studies will be needed to confirm these findings and assess their generalizability to other geographic regions.

This study confirmed that HPV53 can be classified into four major lineages (A-D), with lineage D further subdivided into four sublineages (D1-D4), consistent with previous reports [20]. The 89 novel isolates characterized in this study were distributed across all lineages except lineage B, which has previously been reported only in Rwanda. Although these isolates were obtained from Yunnan province, China, their distribution across the remaining lineages suggests reasonable representation of the broader sublineage diversity of HPV53 currently in circulation. The identification of sublineage D3, composed exclusively of sequences from this study, suggests a previously uncharacterized locally circulating variant. The intermediate mutational profile of isolate 53YN32 bridges sublineages D2 and D3, offering insights into ongoing evolutionary divergence and the history of HPV53 within the Chinese population.

Genome-wide analysis of 89 novel HPV53 isolates revealed an overall nucleotide variability of 4.4%, with the URR exhibiting the highest variability among all genomic regions, consistent with its role as a regulatory sequence subject to less functional constraint than coding regions [31]. Compared to the L1 and L2 regions, the early viral protein regions showed higher variability. Among the coding regions, E2 was the most variable protein (9.1% amino acid differences), while E7 was the most conserved. Distinct lineage- and sublineage-specific nucleotide substitution patterns were identified across lineages A, C, and D, while several amino acid substitutions were conserved across all sublineages, including T77I in E1 and N25A, S149P, S153N, and V340A in E2. Selection pressure analysis revealed that none of these sites was statistically identified as being under positive selection. The consistent presence of these substitutions across diverse isolates suggests they may represent adaptive mutations that confer a selective advantage, potentially through effects on viral replication efficiency, protein stability, or immune evasion. Whether these variants influence MHC binding affinity or T-cell recognition, as has been demonstrated for variants in E6 and E7 of other high-risk HPV types [15], warrants further functional investigation.

Recent evidence indicates that, beyond the classical E6 and E7 oncoproteins, E1 and E2 are emerging as promising therapeutic vaccine targets due to their roles in cervical malignancy [32,33]. Based on this rationale, immunoinformatic analysis identified 176 HLA class I-restricted T-cell epitopes across these four proteins. Several predicted epitopes were consistent with our prior findings [34], such as LLDGNPVSL (A02-E1) and MLMGTVELV (A02-E7-1), both of which have been experimentally validated. Analysis of amino acid substitution effects revealed that sequence variation can substantially modulate epitope immunological properties (Table S10). For instance, substitutions at positions 79 (S-to-P) and 85 (I-to-M) in E7 markedly decreased IEDB PR values, suggesting enhanced HLA-binding affinity. Conversely, a substitution at E1 position 77 (T-to-I) converted a non-antigenic epitope to an antigenic one. These findings underscore the complex effects of sequence variation on immunogenicity and highlight the importance of surveillance studies on currently circulating variants.

From the 176 predicted epitopes, 20 candidates restricted by four HLA-I alleles with high population coverage were selected for experimental validation. ELISA-based peptide-exchange assays confirmed strong binding for 8 peptides across the four alleles tested, and the T2 cell-based stabilization assay further confirmed strong HLA-A*02:01 binding for A02-E7-1, A02-E2-2, and A02-E1. Partial discordance between the two assay systems likely reflects differences in the biological processes they capture: the ELISA-based assay evaluates intrinsic peptide-MHC binding capacity, whereas the T2 cell assay additionally captures MHC stabilization at the cellular level. These results reinforce that combining complementary validation methods is necessary to overcome the limitations of any single assay, and that computational predicted results must be experimentally validated.

The murine immunization experiments demonstrated that only the E6 peptide pool induced robust IFN-γ responses, while E7, E1, and E2 pools elicited only low-level responses. This was unexpected given that E7 is typically considered a primary immunogenic target in therapeutic HPV vaccine development [35]. Two explanations may account for this observation. First, the 13 peptides selected for murine immunization were from the 20 peptides that were selected based on human MHC- related parameters and may therefore not represent optimal antigens for the murine MHC system. Divergent peptide-binding and presentation pathways between human HLA and murine MHC can lead to discrepancies between predicted epitopes and actual murine immune responses [36]. Second, the immunodominance of E6 may reflect intrinsic properties of the HPV53 E6 protein that render it particularly amenable to antigen processing and presentation in this experimental context. Similarly, preclinical evaluation of an HPV-11 DNA vaccine revealed a comparable striking superiority of E6-derived epitopes over E7 [37]. This divergence in CD8^+^ T-cell immune responses can be attributed to the distinct efficiencies in their antigen processing and MHC class I presentation pathways [38]. These findings highlight a fundamental challenge in preclinical vaccine research: the differences between human HLA molecules and murine MHC molecules limit the direct translatability of murine immunization results, underscoring the need for future validation in human T-cell assays or non-human primate models.

Among the E6-drived peptides, A24-E6 (VYNFAYTDL) emerged as the immunodominant T-cell epitope, inducing significantly stronger IFN-γ responses than all other tested E6 peptides. This epitope also demonstrated high binding affinity for HLA-A*24:02, an HLA allele has a higher distribution in the Chinese population and globally [22,23], thereby enhancing its translational relevance for vaccine development targeting this demographic. Homology analysis further revealed conservation of A24-E6 across 12 high-risk HPV genotypes, suggesting that this epitope may elicit broad-spectrum T-cell responses capable of targeting multiple HPV types. However, immunogenicity in this study was assessed by IFN-γ production as a proxy for T-cell activation, without directly measuring cytotoxic killing activity. Future investigations incorporating cytotoxicity assays and multifunctional T-cell analyses are warranted to fully elucidate the therapeutic potential of these validated epitopes.

## 5. Conclusions

In summary, this study provides a comprehensive characterization of the genetic diversity of HPV53 and identifies several experimentally validated T-cell epitopes with strong binding affinity and robust immunogenicity. The conservation of several validated epitopes across multiple high-risk HPV genotypes further suggests potential for broader cross-reactive immunity. These findings provide critical insights into HPV genotypes not currently covered by prophylactic vaccines and lay a foundation for the design of HPV53-targeted therapeutic vaccines.

## Figures and Tables

**Table 1:** Primers for HPV53 whole genome sequencing.

Primer Name	Upstream Primer/Reverse Primer	Product Size (bp)
53Primer1-F	5′-TAACTGCAATGGCGTCACCT-3′	3095
53Primer1-R	5′-GCATATATGGCGTGTGTGGT-3′	
53Primer2-F	5′-AAACGTCCCAGAACCACAGA-3′	3172
53Primer2-R	5′-CTGATTGTTCCAACAGATGCCA-3′	
53Primer3-F	5′-ATGGTGGACACAGGTTTTGGT-3′	2960
53Primer3-R	5′-TACCTGTTGCGTTGCTTCCA-3′	

**Table 2:** Genomic diversity of the 89 novel HPV53 isolates identified in this study.

Gene	Max Nucleotide Pairwise Difference	Nucleotide Sequence Length (bp)	Number of Variable Nucleotide Positions		Max aa Pairwise Difference	Amino Acid Sequence Length (aa)	Number of Variable aa Positions	
			Number	%			Number	%
E6	3.7%	465	28	6.0	5.2%	154	9	5.8
E7	1.3%	318	6	1.9	2.8%	105	5	4.8
E1	1.3%	1911	66	3.5	1.9%	636	29	4.6
E2	2.1%	1155	61	5.3	4.4%	384	35	9.1
L2	2.2%	1392	53	3.8	1.7%	463	16	3.5
L1	2.1%	1500	64	4.3	1.6%	499	17	3.4
URR	4.4%	818	64	7.8	-	-	-	-
CG	1.7%	7859	342	4.4	-	2476	111	4.5

URR, upstream regulatory region; CG, complete genome; aa, amino acid.

**Table 3:** Characterization of conserved CTL candidate epitopes for vaccine validation.

Human Allele	Epitope	Sequence	Length	PR	VaxiJen Score	Total Score	MHC IC50
HLA-A*0201	A02-E6	RTLHQLCEV	9	0.98	0.5368	−0.72	102.4
	A02-E7-1	MLMGTVELV	9	0.06	0.8104	0.57	4.1
	A02-E7-2	QMLMGTVEL	9	0.24	0.6432	0.31	47.7
	A02-E1	LLDGNPVSL	9	0.02	0.784	0.29	49.3
	A02-E2-1	KIPPSVSLV	9	0.15	0.8344	−1.07	145
	A02-E2-2	FLDIVKIPPSV	11	0.14	0.4636	−0.85	74.9
HLA-A*1101	A11-E6-1	ASLEALTKK	9	0.01	0.6362	−0.16	18.6
	A11-E6-2	LLFYSKVRK	9	0.39	0.4773	−0.41	49.5
	A11-E7	QLAVQSSTK	9	0.78	0.7646	−1.4	281.3
	A11-E1	AVALYFYK	8	0.58	0.461	−0.52	35.3
	A11-E2-1	ASSSLRPGK	9	0.07	1.0461	−0.42	34.5
	A11-E2-2	KTAPVVHIK	9	0.01	1.7273	0.14	11.2
HLA-A*2402	A24-E6	VYNFAYTDL	9	0.22	1.1953	−0.68	196.7
	A24-E1-1	RYQGVEFISF	10	0.01	1.0601	1.68	7.3
	A24-E1-2	VYQLNNANW	9	0.02	1.1447	0.27	35.1
	A24-E2	IYCPDSVSSTF	11	0.02	0.4858	0.23	163.3
HLA-B*0702	B07-E6	RPRTLHQL	8	0.02	0.9593	0.55	20
	B07-E7	AVQSSTKEL	9	0.63	0.4585	−0.99	1040.6
	B07-E1	KIRSPAVAL	9	0.03	0.4428	0.9	15.1
	B07-E2	IPPSVSLVL	9	0.09	0.9339	−0.33	169.2

CTL, Cytotoxic T Lymphocyte; PR, Percentile Rank.

## Data Availability

The original data presented in the study are openly available in GenBank under accession numbers PX685428-PX685504 and PZ161758-PZ161769.

## Supplementary Materials

The following supporting information can be downloaded at https://www.mdpi.com/article/10.3390/microorganisms14071395/s1: Table S1: List of HPV53 complete genomes from GenBank used in this study; Table S2: Twenty-three selected HLA alleles with high distribution frequencies worldwide and in China; Table S3: Sequences of positive and negative peptide controls; Table S4: Clinical characteristics of 158 HPV53-positive samples; Table S5: Differences among HPV53 lineages and sublineages (mean ± standard deviation); Table S6: Variations across the 89 complete HPV53 genome sequences; Table S7: Potential HLA class I T-cell epitopes of HPV53; Table S8: Thirteen peptides used in murine immunization experiments; Table S9: Homology analysis between validated epitopes and other 13 high-risk HPV sequences; Table S10: Representative examples of amino acid variations leading to changes in predicted T-cell epitopes between the HPV53 reference sequences and variants; Figure S1. Recombination detection and verification of HPV53 isolate Seq48 using RDP4; Figure S2. SimPlot similarity plot of recombinant isolate Seq48 versus two parental lineages. References [39,40,41,42] are cited in the Supplementary Material.

## Abbreviations

The following abbreviations are used in this manuscript:

HPV	Human papillomavirus
HR-HPV	High-risk human papillomavirus
ORFs	Open reading frames
MHC	Major histocompatibility complex
CTL	Cytotoxic T lymphocyte
DNA	Deoxyribonucleic acid
PCR	Polymerase chain reaction
IEDB	Immune Epitope Database
HLA-I	HLA class I
FI	Fluorescence index
MFI	Mean fluorescence index
FBS	Fetal bovine serum
IFN-γ	Interferon-γ
SFCs	Spot-forming cells
SD	Standard deviation
LSIL	Low-grade squamous intraepithelial lesion
HSIL	High-grade squamous intraepithelial lesion
URR	Upstream regulatory region
SNPs	Single nucleotide polymorphisms
C	Cytosine
T	Thymine
G	Guanine
P	Proline
S	Serine
PR	Percentile Rank
