# Supplementary figures and images for: Genetic Variation of HPV53 and the Identification of T-Cell Epitopes
Source: Microorganisms. 2026 Jun 24;14(7):1395. doi: 10.3390/microorganisms14071395 (PMC13414063; doi:10.3390/microorganisms14071395)

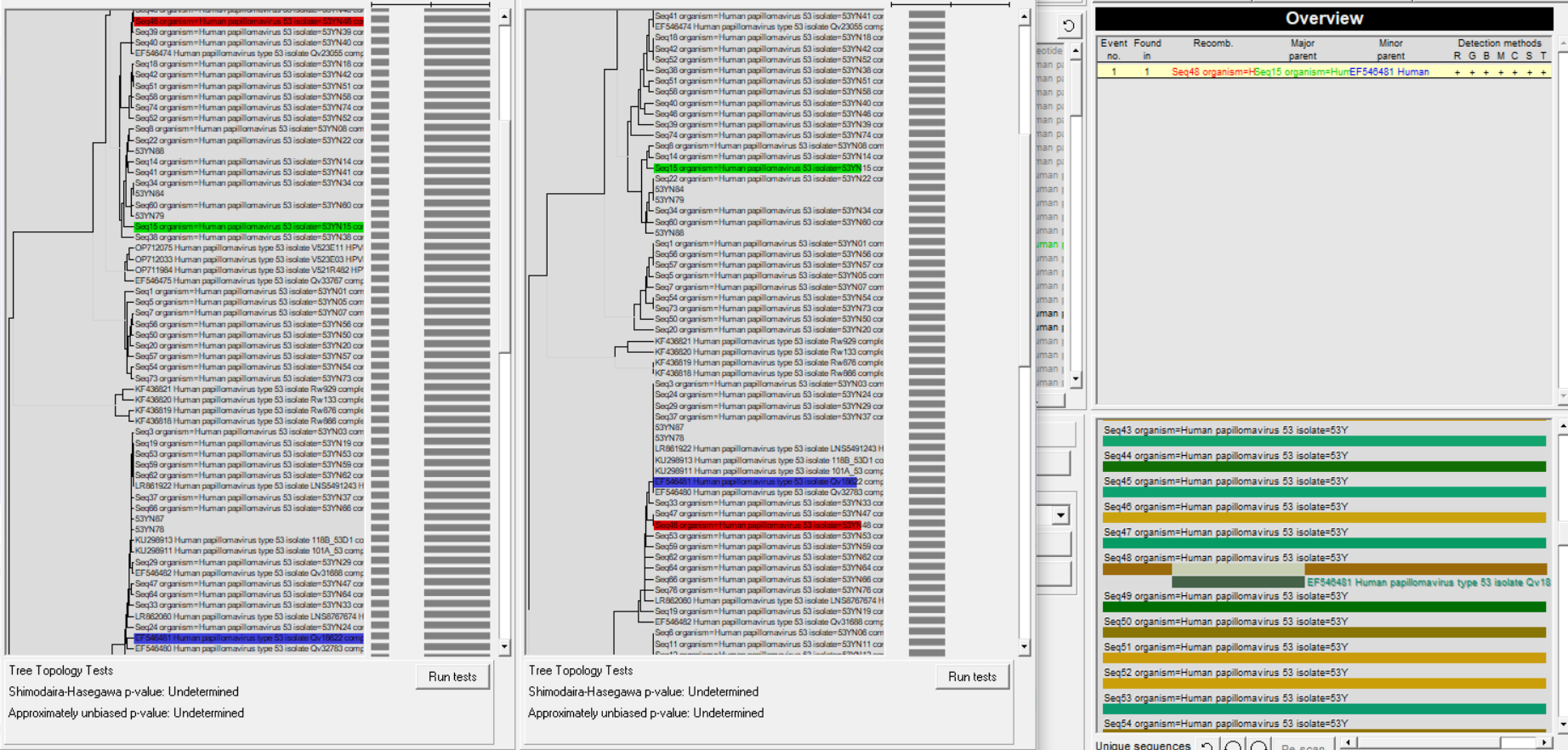

Supplement: Supplementary file 1 [file microorganisms-14-01395-s001.zip › Figure S1. Recombination detection and verification of HPV53 isolate Seq48 using RDP4 .png]

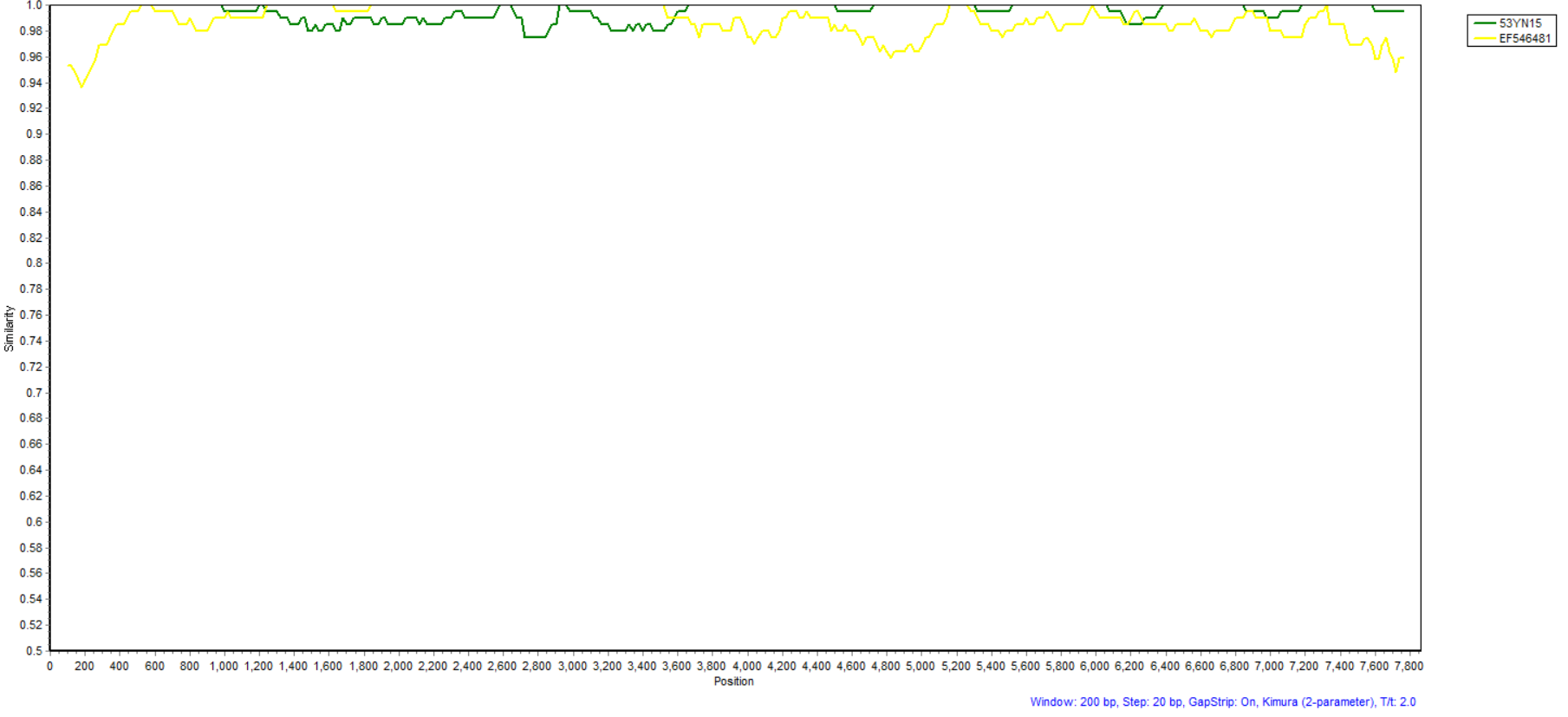

Supplement: Supplementary file 1 [file microorganisms-14-01395-s001.zip › Figure S2. SimPlot similarity plot of recombinant isolate Seq48 versus two parental lineages.png]
